# A case report of anti-GAD65 antibody-positive autoimmune encephalitis in children associated with autoimmune polyendocrine syndrome type-II and literature review

**DOI:** 10.3389/fimmu.2023.1274672

**Published:** 2023-11-22

**Authors:** Tamang Sapana, Wei Li, Fengyan Tian, Wenhao Yan, Binghua Dou, Shuang Hua, Zhihong Zhuo

**Affiliations:** ^1^Department of Pediatric, The First Affiliated Hospital of Zhengzhou University, Zhengzhou, Henan, China; ^2^Department of Pediatric Endocrinology, The First Affiliated Hospital of Zhengzhou University, Zhengzhou, Henan, China

**Keywords:** anti GAD65 antibody, T1DM (type 1 diabetes mellitus), autoimmune thyroiditis, pediatric, autoimmune polyendocrine syndrome (APS type 2), abnormal (behavior), autoimmune encephalitis

## Abstract

**Background:**

Glutamic acid decarboxylase (GAD) is the rate-limiting enzyme for the synthesis of gamma-aminobutyric acid (GABA), the major inhibitory neurotransmitter in the central nervous system. Antibodies against glutamic acid decarboxylase (GAD) are associated with various neurologic conditions described in patients, including stiff person syndrome, cerebellar ataxia, refractory epilepsy, and limbic and extra limbic encephalitis. While there are few case reports and research on anti-GAD65 antibody-associated encephalitis in adults, such cases are extremely rare in pediatric cases.

**Methods:**

For the first time, we report a case of anti-GAD65-positive autoimmune encephalitis associated with autoimmune polyendocrine syndrome (APS) type II. We reviewed previously published pediatric cases of anti-GAD65 autoimmune encephalitis to discuss their clinical features, laboratory tests, imaging findings, EEG patterns, and prognosis.

**Case presentation:**

An 8-year-old, male child presented to the outpatient department after experiencing generalized convulsions for twenty days. The child was admitted for epilepsy and had received oral sodium valproate (500 mg/day) in another center, where investigations such as USG abdomen and MRI brain revealed no abnormalities, however, had abnormal EEG with diffuse mixed activity in the left anterior middle prefrontal temporal region. On the follow-up day, a repeat blood test showed a very low serum drug concentration of sodium valproate hence the dose was increased to 750 mg/day. Then, the child experienced adverse effects including increased sleep, thirst, and poor appetite, prompting the parents to discontinue the medication. A repeat MRI showed increased signals on FLAIR sequences in the right hippocampus hence admitted for further management. The child's past history included a diagnosis of hypothyroidism at the age of 4, and receiving levothyroxine 75 mcg once daily. His parents are healthy with no history of any similar neurological, autoimmune, or genetic diseases, but his uncle had a history of epilepsy. At presentation, he had uncontrolled blood glucose levels with elevated HbA1c levels. Additionally, the serum and CSF autoantibodies were positive against the anti-GAD65 antibody with the titer of 1:100 and 1:32 respectively. The patient was managed with a mixed type of insulin regimen and received first-line immunotherapy (intravenous immunoglobulin, IVIG) for five consecutive days, followed by oral prednisone and sodium valproate as an antiepileptic drug. Upon achieving a favorable clinical outcome, the patient was discharged with oral medications.

**Results:**

Among the 15 pediatric patients reported in this literature, nine presented with limbic encephalitis (LE), three with extralimbic encephalitis (ELE), and three with a combination of limbic and extralimbic encephalitis. Most of these cases exhibited T2-W FLAIR hyperintensities primarily localized to the temporal lobes in the early phase, progressing to hippocampal sclerosis/atrophy in the later phase on MRI. EEG commonly showed slow or spike waves on frontotemporal lobes with epileptic discharges. Prognostic factors varied among patients, with some experiencing persistent refractory seizures, type-1 diabetes mellitus (T1DM), persistent memory impairment, persistent disability requiring full assistance, and, in severe cases, death.

**Conclusion:**

Our findings suggest that anti-GAD65 antibody-positive autoimmune encephalitis patients may concurrently present with other APS. Our unique case presented with multiple endocrine syndromes and represents the first reported occurrence in children. Early diagnosis and timely initiation of immunotherapy are crucial for improving clinical symptoms and reducing the likelihood of relapses or permanent disabilities. Therefore, emphasis should be placed on prompt diagnosis and appropriate treatment implementation to achieve better patient outcomes.

## Introduction

Autoimmune encephalitis is a neurological disorder, characterized by confusion, memory disturbances, and often seizures (1). Anti-GAD65 encephalitis is subtype of autoimmune encephalitis. Reported cases of anti-GAD65 encephalitis in pediatric patients have been associated with a single endocrine syndrome whereas our case is a novel case of anti-GAD65 encephalitis in a pediatric patient which concurrently involve with multiple endocrine syndromes (T1DM and autoimmune thyroiditis). This particular case is the rare combination that has been observed in a young patient and the first case of its kind to have ever been reported.

To the best of our knowledge, only a single type of autoimmune endocrine illness has been associated with anti GAD65 in pediatrics in previously published papers. This work has so far concentrated on autoimmune encephalitis with anti-GAD65 and multiple autoimmune endocrine disorders. Additionally, we aimed to raise awareness of anti-GAD-65-mediated autoimmune encephalitis, which can appear in conjunction with a number of endocrine disorders. Anti-GAD-65 antibody testing should be taken into consideration to individuals who exhibits any symptoms suggestive of autoimmune encephalitis, and strict monitoring for the emergence of T1DM and other autoimmune endocrine illnesses is advisable. Early recognition and timely management of such cases may improve patient outcomes and help researchers better understand the intricate relationships between the nervous and endocrine systems in autoimmune encephalitis.

## Case presentation

**Figure 1:**
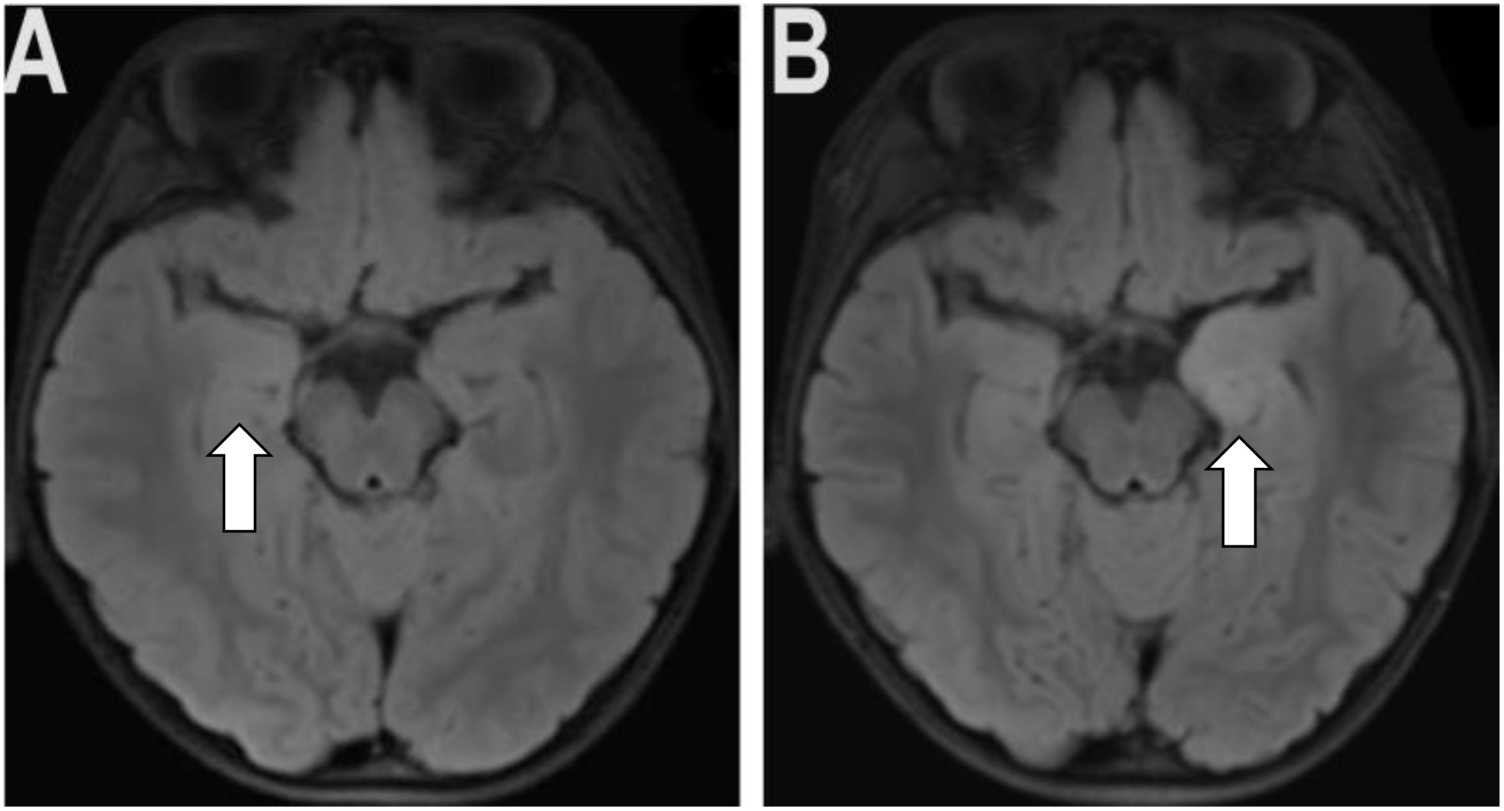
**(A)** showing an increased signal on the FLAIR sequence of the right hippocampus. **(B)** showing the new inflammatory lesion on the left hippocampal. In addition, it demonstrates that the original right hippocampal signal was decreased with compared to the old film.

An 8-year-old, male child presented to the outpatient department after experiencing generalized convulsions for twenty days. The child was admitted for epilepsy and had received oral sodium valproate (500 mg/day) in another center, where investigations such as USG abdomen and MRI brain revealed no abnormalities. However, VEEG (video electroencephalogram) indicated abnormal EEG with diffuse mixed activity (ranging from low to high amplitude sharp waves, spicy slow waves/sharp slow waves) of 1.5-5.5 Hz in the left anterior middle prefrontal temporal region. On the follow-up day, a repeat blood test showed a very low serum drug concentration of sodium valproate hence the dose was increased to 750 mg/day. However, the child experienced adverse effects including increased sleep, thirst, and poor appetite, prompting the parents to discontinue the medication. A repeat MRI showed increased signals on FLAIR sequences in the right hippocampus (**Figure 1A**) hence admitted for further management.

The child's past history included a diagnosis of hypothyroidism at the age of 4, and receiving levothyroxine 75 mcg once daily. His parents are healthy with no history of any similar neurological, autoimmune, or genetic diseases, but his uncle had a history of epilepsy.

## Diagnosis and treatment

Our patient exhibited various clinical and laboratory abnormalities leading to the diagnosis of anti-GAD65 autoimmune encephalitis associated with APS type-II. Initial hematological tests showed normal results except for hyperglycemia (20.72 mmol/L) and increased glycosylated hemoglobin (HbA1c) to 9.2%, indicating uncontrolled T1DM. Urine analysis revealed the presence of ketone bodies (3+) and glucose (3+), along with elevated levels of glycosylated serum protein and D3 hydroxybutyric acid, confirming diabetic ketoacidosis.

Further investigations showed low fasting C-peptide levels, and elevated anti-insulin and anti-islet cell antibodies, indicating a deficiency in pancreatic beta cell insulin production, leading to hypoinsulinemia, and diagnosing autoimmune-mediated T1DM. In order to control blood sugar levels, the patient was put on an insulin regimen. The maximum blood sugar recorded was 28.3 mmol/L, and to achieve a normal blood glucose level, the dose of the mixed type of insulin regimen (long/ short-acting) was adjusted accordingly.

Cerebrospinal fluid (CSF) analysis demonstrated elevated glucose levels and increased CSF IgG (133 mg/L) with the presence of oligoclonal bands in CSF, confirming autoimmune brain disease. Other CSF analysis revealed no pleocytosis with normal CSF and serum albumin (116.70mg/L and 43.70g/L). The albumin quotient value of 2.67x10^-3^ (Normal range- 0-9x 10^-3^) which was calculated by (QAlb = CSF albumin/serum albumin) (2), indicating normal the blood-CSF barrier function. Rheumatological tests revealed positive results for antinuclear antibody (ANA), anti-SSA-52 antibody, further supporting the autoimmune nature of the disorder.

**Figure 2:**
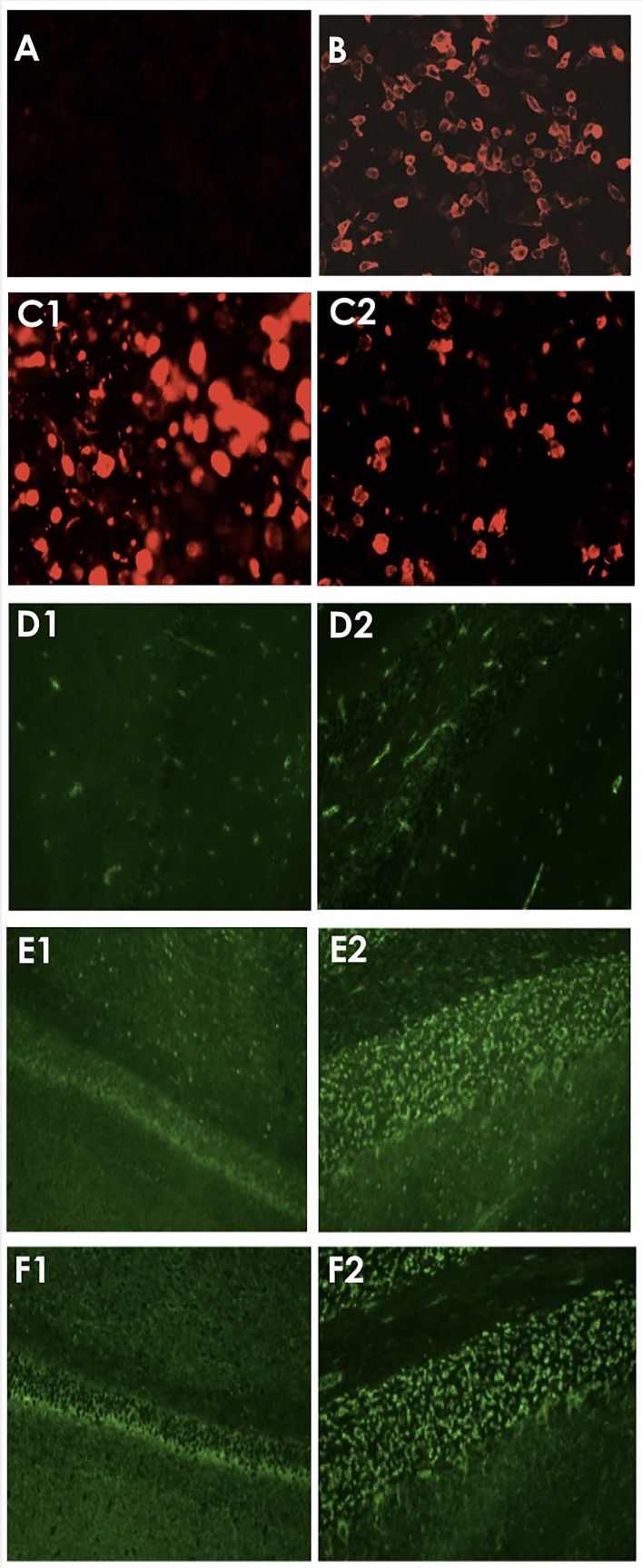
Test results of serum and CSF using indirect immunofluorescence assays. **(A, B)** showed negative and positive controls of CBA technique respectively. Red fluorescent light showing positive results detected by CBA in serum **(C1)** and CSF **(C2)**. **(D1, D2)** illustrated the negative controls detected by TBA method in hippocampus and cerebellum respectively. While **(E1, E2)** in green fluorescent light showing the positive TBA in hippocampus and cerebellum tissue respectively by using serum, and **(F1, F2)** showing positive results of TBA by using CSF.

Increase level of serum GAD antibody (160.92 IU/mL/ Normal value- 0-10 IU/mL) indicating the presence of anti-GAD antibody. Furthermore, the anti-GAD65 antibody titer was detected using indirect immunofluorescence cell-based assays (CBA) in both serum and CSF with titers of 1:100 and 1:30 respectively. The brain tissue slices detected by tissue-based assay (TBA) showed positive results (**Table 1**; **Figure 2**) in both serum and CSF. First MRI of the brain revealed increased signals in the right hippocampus, suggesting brain involvement (**Figure 1A**).

**Table 1:** Autoimmune encephalitis panel.

Test	Detection Method	Results	
		Serum	CSF
NMDA- IgG AMPA1-IgG AMPA2-IgG LGI-1-IgG CASPR2-IgG GlyR1-IgG GABA-A GABA-B-IgG IgLON5-IgG DPPX-IgG DRD2- IgG GAD65-IgG mGluR1-IgG mGluR5-IgG Neurexin-3 α Ganglionic AchR KLHL11 GluK2 AK5 AG0 CaVα2δ AQP4 MOG GFAP TBA detection of Brain tissue slices	CBA CBA CBA CBA CBA CBA CBA CBA CBA CBA CBA CBA CBA CBA CBA CBA CBA CBA CBA CBA CBA CBA CBA CBA TBA	Negative Negative Negative Negative Negative Negative Negative Negative Negative Negative Negative Positive (1:100) Negative Negative Negative Negative Negative Negative Negative Negative Negative Negative Negative Negative Positive	Negative Negative Negative Negative Negative Negative Negative Negative Negative Negative Negative Positive (1:30) Negative Negative Negative Negative Negative Negative Negative Negative Negative Negative Negative Negative Positive

The 24 autoimmune encephalitis panels in both serum and cerebrospinal fluid (CSF).

The results were detected by indirect immunofluorescence cell-based assay (CBA) indicating that the anti-GAD65 antibodies were positive in both serum and CSF with titers of 1:100 and 1:30 respectively. The brain tissue slices detected by tissue-based assay (TBA) were positive.

The patient's thyroid function test showed low free T3 and high thyroid-stimulating hormone (TSH), along with elevated anti-thyroid peroxidase(198 IU/mL) and anti-thyroglobulin antibodies (>4000 IU/mL) in serum, confirming autoimmune thyroiditis. After confirming the presence of positive anti-GAD65 antibodies, T1DM, and autoimmune thyroiditis, a diagnosis of anti-GAD65 antibody autoimmune encephalitis associated with APS type-II was made.

The results of various tests, including serum lactic acid, blood ammonia level, cardiac enzymes, T-SPOT test, parathyroid hormone level, immunoglobulin complement tests, infectious disease screening, and iron profile, did not show any significant abnormalities. However, ACTH and Cortisol levels measured in the morning and evening were within normal range. Additionally, ultrasound scans for abdominal and testicular masses, as well as serum and CSF screening for paraneoplastic conditions, yielded negative findings. The homocysteine test in combination with folic acid was normal, but vitamin B12 levels were elevated. Furthermore, both cardiac echo and plain chest CT scan results were normal, indicating the absence of any cardiovascular risks.

The patient was treated with human immunoglobulin (IVIG) (2 gm/kg/day) as the first-line immunotherapy for five consecutive days, followed by oral prednisone 45 mg/day (2 mg/kg/day). The insulin regimen was readjusted to achieve optimal blood glucose levels. The child showed improvement in sleep patterns, appetite, and seizure frequency after the treatment. A follow-up MRI showed a decrease in the inflammatory lesion on the right hippocampus and the appearance of a new inflammatory lesion on the left hippocampus. (**Figure 1B**).

The patient exhibited clinical improvement after 10 days of therapy, and was discharged with medications (prednisone 45 mg/day, levothyroxine 50 mcg per day, sodium valproate and mixed type insulin regimen pump) with instructions for monitoring blood glucose levels at home.

The child developed a left sided headache five months after being discharged, which was acute in nature and was followed by generalized seizures that lasted for around 30 minutes. His MRI revealed no new lesions and diminished existing lesions. VEEG showed aberrant waves of moderate to high amplitude sharp slow waves, intermittent slow waves, and intermittent left and right-synchronous or asynchronous bursts in bilateral frontal, central, parietal, and occipital regions during sleep. Furthermore, within 24 hours of the VEEG, two clinical seizures were discovered. Repeat IVIG (2 gm/kg) was given for 5 days, followed by 50 mg of prednisone, orally. To control the seizures, a fresh addition of tablet lacosamide (100 mg in the morning and 50 mg in the evening) was made and stopped sodium valproate. Nevertheless, continuing anti-thyroid drugs and insulin pump as directed. With these treatment the child was clinically recovered.

## Discussion

Autoimmune polyendocrine syndrome type-II (APS II) also known as Schmidt’s syndrome, is a rare condition characterized by the co-occurrence of at least two of the following endocrine disorders: primary adrenal insufficiency (Addison’s disease), primary hypothyroidism, type 1 diabetes mellitus, celiac disease, and pernicious anemia (3, 4). APS-II is infrequent among children and any patient with Addison’s disease (AD) has a 50% lifetime risk of developing additional autoimmune disease. It is strongly recommended to conduct periodic retesting at intervals of 2-3 years, as autoantibodies have the potential to develop at any point throughout an individual’s lifespan (5).

In our case, the patient presented with positive anti-GAD65 antibody, anti-islets cells, and anti-insulin antibody along with positive anti-thyroid peroxidase antibody and anti-thyroglobulin antibody, with more than two autoimmune diseases confirmed the diagnosis of APS type-II. This represents the first-ever reported case of APS-II with anti- GAD antibody autoimmune encephalitis in the pediatric age group.

The presence of anti-GAD65 antibodies is frequently associated with T1DM, and these antibodies can be detected in a significant proportion of patients with newly-diagnosed T1DM and prediabetic individuals (6). Additionally, anti-GAD-65-mediated autoimmune encephalitis, characterized by non-paraneoplastic intracellular antigens, demonstrates pathogenicity to the central nervous system (7).

Several cases have been reported regarding anti-GAD65 antibodies in this literature (summarized in **Table 2**), the first case being reported in 2002 (8). In our study, we present a comprehensive analysis of 15 cases, including our own, involving patients under the age of 18. We found similarities with the work of Ren et al. (2021) (17), who reported 10 cases of anti-GAD65 antibody-associated encephalitis. Among all cases, nine exhibited features of limbic encephalitis (LE), three had extralimbic encephalitis (ELE), and three presented a combination of limbic and extralimbic encephalitis.

**Table 2:** Pediatric cases of anti-GAD65 antibodies associated with encephalitis in previous studies from 2002 to 2022.

Author/Year	Age/Sex	Clinical features	Autoimmune disorder	GAD Ab titer		OCB	Brain MRI	EEG	Immunotherapy	Prognosis
				Serum	CSF					
1.Olson et al (8)	6y/M (2002)	Epilepsia partialis continua, aphasia	Type 1 Diabetes Mellitus	19610 U/ml (normal range < 1.0 U/ml)	3325 U/ml (normal range < 1.0 U/ml)	Not mentioned	Initially: normal Later: lesions of the gray matter involving the occipital and frontal cortex, left insular region, cerebellum	Left-sided epileptiform discharges and slowing	High-dose steroids, IVIG, PE	Seizure free
2. Akman et al (9)	16y/Fe (2009)	Complex partial seizure and status epilepticus, academic function decline	Common variable immune deficiency (CVID)	>300 IU/ml (normal range 0–1.45)	>300 IU/ml (normal range 0–1.45)	Not mentioned	Initially: bilateral hippocampal T2 hyperintensities 1 year later: bilateral mesial temporal sclerosis	Bilateral temporal onset complex partial status epilepticus	MP, IVIG	Refractory seizures
3.Korff et al (10)	6y/Fe (2011)	Progressively refractory focal seizures, progressive global developmental delay, and gait instability.	T1DM at 3 years of age	3400 IU/mL (Normal-<10 IU/mL)	13 IU/m (Normal-<1IU/mL)	Positive	Initially: normal at 5 years: 3yrs Later: bilateral hippocampal, cortical, and cerebellar atrophy	Multifocal discharges and right frontal seizures	MP, Plasma exchange, mycophenolate mofetil, rituximab	Clinical improvement but had refractory seizures
4.Navin et al (11)	15y/M (2014)	Headache, transient memory disturbance, seizures, behavior change.	None	1:160000 (CBA)	1:128000 (CBA)	Positive	T2/FLAIR signal of the right hippocampus and amygdala, mildly increased signal in the left amygdala	Interictal epileptiform discharges arising independently from the right frontotemporal and left the posterior head region	IVIG, oral prednisone, RTX	Excellent seizure control; improvement of transient global amnesia-like episodes
5. Incecik et al (12)	7y/M (2015)	Behavioral changes, dysphagia, ptosis, diplopia, and drowsiness	None	Positive	Positive	Not mentioned	Normal	Epileptiform abnormalities in both temporal regions	IVIG, Plasma exchange	Complete recovery
6. Grilo et al (13)	10y/Fe (2016)	Headache, nausea, vomiting, seizures, irritability, depressed mood, unusual fear, memory disturbance Later: new seizures, decrease the state of consciousness, confusion, hallucination	Type-1 Diabetes mellitus	2793 U/mL (Normal-<1 U/mL)	Positive	Negative	Hypertense signal of the left mediotemporal lobe and amygdala on T2 and FLAIR sequences	Paroxysmal activity and Bilateral temporal slowing	MP, Oral prednisone, RTX	Improved (No seizures and neuropsychological deterioration)
7. Achour et al (14)	9y/Fe (2017)	Temporal lobe seizures, generalized tonic-clonic seizures, Mood and behavioral disturbances, autonomic imbalance.	None	Positive	Positive	Not mentioned	Normal	Slowed theta rhythm with bilateral frontotemporal spike-wave discharges	MP, IVIG, RTX	6 months: died
8.Akin et al (15)	16y/M (2017)	Confusion and headache	Type-1 DM 6months back	2114 IU/mL (Normal-0-10)	4.07 nmol/L (Normal<0.02)	Not mentioned	Hyperintense signal of the right mesiotemporal lobe	Temporal slowing	IVIG,MP, Oral steroid	Persists with type-1 DM
9. Koki et al (16)	5y/Fe (2018)	Seizure clustering and altered mental status	Pineoblastoma (Onco)	Initially:65,100 U/mL (Normal -<1.5 U/mL) Later: 1,42,000 U/mL	Initially: not mentioned Later: 238 U/mL	Not mentioned	Initially: Hyperintensity of cerebrum, predominantly in the left hemisphere. Later: severe whole brain encephalitis	Initially: left dominant delta activity. Later: generalized delta activity and no spike waves.	IVIG, MP, Plasma exchange Tacrolimus	Still unable to communicate and walk independently and requires almost total assistance.
10. Kern et al 3	16y/Fe (2021)	Persistent headache, 2 episodes of urinary incontinence, change in acute mental status, expressive aphasia, auditory hallucination.	Acute lymphoblastic Leukemia(0nco)	>250 IU/mL (Normal <5 IU/mL)	Negative	Positive	New edema and ill-defined enhancement involving the left temporal lobe, parieto-occipital region, and left hippocampus	Subclinical seizures from the left frontal, temporal lobe.	IVIG, Dexamethasone	Symptomatic improvement but persists Type-1 DM
11. Ren et al (17)	6y/Fe (2021)	Seizure, headache, memory deficient	None	1:100(CBA)	1:320(CBA)	Positive	Bilateral Hippocampus	Right-sided epileptiform discharge	IVIG, MP, Oral steroids	Refractory focal seizures
12. Ren et al (17)	16y/Fe (2021)	Seizure, memory deficient, depression, dysautonomia	Thyroiditis	1:32(CBA)	1:32(CBA)	Positive	Initial: bilateral hippocampal 15th month: bilateral frontal lobe, left parietal lobe, right temporal lobe and insular cortex, and subcortical white matter and the bilateral hippocampus 5 years follow-up: parenchymal atrophy.	Slowed theta rhythm with bilateral temporal spike-wave discharges	IVIG, MP, oral steroid, RTX	Persistent memory impairment and refractory focal seizures
13. Ren et al (17)	4y9m/F (2021)	Vomiting, headache, confusion	None	1:100(CBA)	1:320(CBA)	Positive	Brainstem, thalamus, basal ganglia, bilateral cerebral, and cerebellar hemispheres	Slow theta rhythm	IVIG, MP, oral steroid	Complete recovery
14. Bushati et al (18)	8y/Fe (2022)	First episode: abrupt behavioral changes, irritability, emotional lability, deficiency of speech, low concentrationation and memory loss, headache, and morning vomiting. Second episode: drop attack, face blindness Third episode: neuropsychiatric complaints	None	Positive (ELISA)	Not mentioned	Not mentioned	Normal	Initially: spiked and slow right frontotemporal alpha waves Later: no clinical seizures were seen	IVIG, MP, RTX	Not mentioned
15.	8y/M (2023)	Seizures increase sleep and thirst and decrease appetite	Hypothyroidism	1:100 (CBA)	1:32 (CBA)	Positive	Initially right hippocampus involvement. Later left hippocampus	Diffuse mixed activity in the left anterior middle prefrontal temporal region	IVIG, MP, Oral prednisone	Persists with Type-1 DM

CBA, cell-based assay, TBA, tissue-based assay, IVIG, intravenous immunoglobulin, MP, methylprednisolone, RTX, rituximab,T1DM, type-1 diabetes mellitus.

Limbic symptoms were prevalent among the patients and included mental and behavioral changes (observed in almost all cases), various types of seizures (12 cases), and memory disturbances (5 cases). The extra limbic symptoms comprised ataxia (one case), aphasia (three cases), headache (seven cases), and global developmental delay (one case). Moreover, some cases experienced autonomic dysfunctions, such as gastrointestinal upsets (nausea, vomiting, dysphagia in five cases), ocular symptoms (ptosis, diplopia both in one case), face blindness (one case), and urinary incontinence (one case). Our patient’s symptoms were characterized by seizures, increased sleep, thirst, and poor appetite.

MRI findings showed T2-W FLAIR hyperintensities primarily restricted to the temporal lobes in the early phase, progressing to hippocampal sclerosis/atrophy in the late phase in majority of the cases. Interestingly, three cases presented with normal MRIs. Likewise, our patient exhibited initial involvement of the right hippocampus, followed by subsequent engagement of the left hippocampus. The majority of cases exhibited the electroencephalogram (EEG) readings of slow waves or spike waves on the frontotemporal lobes, whereas our EEG findings revealed abnormal signals characterized by diffuse mixed activity (ranging from low to high amplitude sharp waves and spicy slow waves/sharp slow waves) in the left anterior middle prefrontal temporal region. But the latest VEEG of our patient elicited mixed type waves with different amplitudes from almost all regions of the brain. These findings contribute to a better understanding of the clinical and neuroimaging characteristics of anti-GAD65 antibody-associated encephalitis in young patients.

The diagnosis of GAD65-Ab-associated encephalitis is based on clinical features and the presence of high anti-GAD65 Ab level in serum or high titers in both serum and CSF with the detection of intrathecal synthesis (IS) in CSF (19). In clinical practice, GAD Ab is detected through different techniques, including indirect immunohistochemistry, immunoblot, enzyme-linked immunosorbent assay (ELISA), and radioimmunoassay (RIA), which have different sensitivity and specificity (20). Recently, CBA technique is considered as an effective screening method. It demonstrates promise as a valuable screening method for suspected patients, TBA plays a significant role in defining antibodies, especially for patients who yield negative CBA results (21). In our patient, we detected high level of GAD antibody in serum along with high titers of anti-GAD65-Abs detected in both serum and CSF through CBA and TBA methods. In addition, 7/15 cases including our case detected oligoclonal bands in CSF, whereas only one case had a negative result, and the remaining cases did not mention.

The first-line treatment for GAD65-Ab-associated encephalitis typically involves IVIG, intravenous methylprednisolone, and plasma exchange. Second-line treatments, such as rituximab, cyclophosphamide, and mycophenolate mofetil, are considered, although their efficacy may vary across cases. Occasionally, third-line therapies, such as daratumumab, bortezomib, tocilizumab, tofacitinib, and low-dose interleukin 6 (IL-6), have been recommended; however, their effectiveness is currently being evaluated in ongoing trials (22). Our patient responded well to the first line of therapy. The prognosis varies for each patient, with early diagnosis and timely immune therapy associated with better outcomes. However, some patients had experienced relapse and require second-line treatments. Persistent refractory seizures, type-1 diabetes mellitus, and other complications (persistent memory impairment, persistent disability and death) have also been reported.

## Conclusion

This represents the initial documentation of a case, presenting anti-GAD65 autoimmune encephalitis in association with APS type-II. Unlike previous reports where anti-GAD65 autoimmune encephalitis coexisted with a single type of endocrine disease, our case illustrates a unique manifestation involving both APS type-II with anti-GAD65 autoimmune encephalitis. The coexistence of three immune diseases (anti-GAD65 antibody, T1DM, and autoimmune thyroiditis) presents a distinctive and uncommon case that holds significant importance in pediatric medicine and in the medical literature, contributing valuable insights for future research. This study aims to increase awareness regarding anti-GAD-65-mediated autoimmune encephalitis, which may concomitantly occur with polyendocrine diseases such as T1DM and autoimmune thyroiditis. Moreover, we believe that early recognition of neuropsychiatric symptoms, coupled with timely initiation of immunotherapy, may yield favorable treatment outcomes and reduce the risk of relapses or permanent disability.

## Data availability statement

The original contributions presented in the study are included in the article/supplementary material. Further inquiries can be directed to the corresponding author.

## Ethics statement

The studies involving humans were approved by The First Affiliated Hospital of Zhengzhou University. The studies were conducted in accordance with the local legislation and institutional requirements. Written informed consent for participation in this study was provided by the participants’ legal guardians/next of kin. Written informed consent was obtained from the individual(s), and minor(s)’ legal guardian/next of kin, for the publication of any potentially identifiable images or data included in this article.

## Author contributions

TS: Writing – original draft. ZZ: Supervision, Writing – review & editing. WL: Data curation, Writing – review & editing. FT: Investigation, Writing – review & editing. WY: Investigation, Writing – review & editing. BD: Investigation, Writing – review & editing. SH: Writing – review & editing, Investigation.
